# Regulation of Neuronal Protein Trafficking and Translocation by SUMOylation

**DOI:** 10.3390/biom2020256

**Published:** 2012-05-14

**Authors:** Anja Berndt, Kevin A. Wilkinson, Jeremy M. Henley

**Affiliations:** School of Biochemistry, Medical Research Council Centre for Synaptic Plasticity, Medical Sciences Building, University of Bristol, University Walk, Bristol, BS8 1TD, UK

**Keywords:** SUMO, protein translocation, receptor trafficking, neuronal excitability, synaptic plasticity

## Abstract

Post-translational modifications of proteins are essential for cell function. Covalent modification by SUMO (small ubiquitin-like modifier) plays a role in multiple cell processes, including transcriptional regulation, DNA damage repair, protein localization and trafficking. Factors affecting protein localization and trafficking are particularly crucial in neurons because of their polarization, morphological complexity and functional specialization. SUMOylation has emerged as a major mediator of intranuclear and nucleo-cytoplasmic translocations of proteins involved in critical pathways such as circadian rhythm, apoptosis and protein degradation. In addition, SUMO-regulated re-localization of extranuclear proteins is required to sustain neuronal excitability and synaptic transmission. Thus, SUMOylation is a key arbiter of neuronal viability and function. Here, we provide an overview of recent advances in our understanding of regulation of neuronal protein localization and translocation by SUMO and highlight exciting areas of ongoing research.

## 1. Introduction

SUMOylation is the covalent attachment of a member of the SUMO family of proteins to one or more lysine residues on target proteins. SUMOylation is best characterized for nuclear proteins involved in genome integrity, nuclear structure and transcription [1,2] but it is now clear that SUMOylation is also important for extranuclear signal transduction, trafficking and modification of cytosolic and integral membrane proteins. Since the first report of SUMOylation as a modification of the nuclear pore component RanGAP [3,4], several hundred SUMOylation substrates have been characterized and many more putative targets have been identified by proteomic studies [5,6].

Neurons are highly-specialized morphologically complex polarized cells that exhibit constant constitutive and activity-dependent directed protein transport. The signaling pathways that orchestrate protein trafficking are necessarily sophisticated and multilayered and it has become evident that for many neuronal proteins SUMOylation is an important factor in regulating their localization and function under both physiological and pathophysiological conditions [7,8]. In this review we focus on recent advances regarding the effects of SUMOylation on protein trafficking in neurons (Table 1).

**Table 1:** Consequences of SUMOylation of proteins of different cell compartments.

Cellular compartment	SUMO substrate	Outcome of SUMOylation
**intranuclear **	PML	formation of PML bodies, possible transcriptional regulation upon axonal damage [9,10]
	BMAL1	association with PML bodies, transcriptional regulation and periodic degradation of BMAL1 [11,12]
**nucleo-cytoplasmic**	GSK3beta	re-localization into the nucleus, enhances stability and stimulates apoptosis [13]
	Caspase 2/7/8	re-localization into the nucleus, possible cleavage of target proteins [14,15,16]
	FAK	re-localization into the nucleus, no functional data yet [17]
**extranuclear**	Arc/Arg3.1	Re-localization into dendrites and to cytoskeleton, important for establishment and maintenance of LTP [18]
	GluK2	internalization of receptor, possible recycling and re-insertion into plasma membrane [19]
	Group III mGluRs	Possible effects on synaptic transmission in the hippocampus, internalization and/or degradation of receptors [20,21]
	CB1	Agonist-induced deSUMOylation potentially regulates internalization of receptor [22]
	La	binding to dynein and retrograde axonal transport [23]

## 2. Regulation of Intra-Nuclear Organization by SUMO

**Figure 1:**
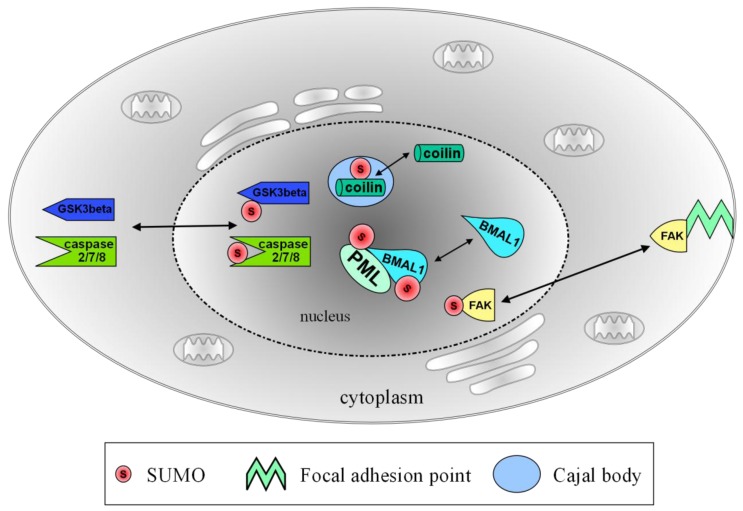
Translocation of nuclear and nucleo-cytoplasmic proteins following SUMOylation. In the nucleus, SUMO modification leads to the formation of Cajal bodies, which are positive for coilin and SUMO. Other SUMO related nuclear structures are PML bodies, which contain SUMOylated PML and SUMOylated BMAL1. Caspases -2/-7/-8, GSK3beta and FAK translocate to the nucleus on SUMOylation. Abbreviations: PML: promyelocytic leukemia; BMAL: brain and muscle aryl hydrocarbon receptor nuclear translocator-like; GSK: glycogen synthase kinase; FAK: focal adhesion kinase.

In the nucleus, SUMOylation is mainly associated with transcriptional regulation and DNA damage repair. SUMO modification of transcription factors or their interaction partners can lead to an increase or decrease in transcriptional activity of the cell [24], while SUMOylation of a number of components of the DNA damage repair machinery is required for their localization to points of DNA damage [25,26]. In neurons, SUMOylation is also emerging as an important regulator of sub-nuclear organization and protein trafficking within the nucleus (Figure 1).

### 2.1. Transient Localization of SUMO and Ubc9 at Cajal Bodies

Cajal bodies (CBs) are small nuclear structures present in metabolically active mammalian cells, especially neurons, where their size is often increased compared to other cell types [27]. They are characterized by the presence of the two main components, the p80 coilin and the survival motor neuron (SMN) protein. CBs are involved in the processing of replication-dependent histone mRNAs and in the biogenesis of ribonucleoproteins associated with pre-mRNA, pre-rRNA processing and splicing [27]. 

CBs are transcription-dependent and high numbers of Cajal bodies are indicative of a transcriptionally active cell with a high cellular mass [28]. SUMO1 and Ubc9 transiently co-localize to Cajal bodies in undifferentiated neuron-like UR61 cells [29]. Although not yet validated as SUMO substrates, both coilin and SMN possess high probability SUMOylation sites and coilin interacts with the SUMO E3 ligase PIASγ [30]. Interestingly, this association occurs only in undifferentiated cells and cells that have been exposed to stress [29]. Therefore, it has been hypothesized that SUMO1 translocation to CBs is dependent on events during neuronal differentiation and forms part of a stress response in differentiated cells. These results are consistent with reports that changes in protein SUMOylation play an important role in neuronal development and survival [8,31,32,33]. Hence, it seems likely that CBs represent a potential target of neuroprotection by SUMOylation.

### 2.2. PML Bodies and Axonal Damage

Another nuclear structure abundant in SUMO is the PML body, which are highly enriched in the promyelocytic leukemia (PML) protein. SUMOylation is prerequisite for the PML body formation since only SUMOylated PML is able to recruit other PML body proteins such as Daxx [9,10,34]. PML bodies are involved in a variety of processes including viral defense, stress response and genome stability [35,36,37]. Although PML bodies appear to be absent from most neurons [38,39] they are present in human dorsal root ganglion neurons (DRGN) [40]. Interestingly, in DRGNs affected by acute inflammatory demyelinating polyneuropathy (AIDP) associated with Guillain-Barre syndrome [41], the number of PML bodies increases with the severity of the dymyelination [40]. Thus, PML bodies are potentially involved in the DRGN response to axonal damage and injury. This is supported by the finding that in AIDP neurons, the glucocorticoid receptor (GR), itself a SUMO substrate, is localized to PML bodies, whereas in healthy cells GR does not localize to any nuclear foci [42,43]. This re-localization might play a role on the transcriptional regulation of genes expressed upon axonal damage.

### 2.3. Keeping the Circadian Rhythm Running: SUMOylation of BMAL1

All organisms are affected by a circadian rhythm, which spans a period of about 24 hours [44]. In mammalians, the circadian rhythm is regulated by a master clock in the suprachiasmatic nuclei of the hypothalamus which is entrained by the natural light-dark cycles [44,45]. Disruption of the circadian rhythm can be involved in bipolar disorder, sleeping disorders and dementia [45]. The transcription factor BMAL1 is an essential component of the clock [44,46] and can be SUMOylated by SUMO1, SUMO2 or SUMO3 [11,12]. Under physiological conditions, BMAL1 is predominantly modified by SUMO2/3, which is involved in the localization of BMAL1 to PML bodies where it functions as a transcription factor [11]. In addition, SUMOylation also triggers ubiquitin-mediated periodic degradation of BMAL1 at PML bodies [11,12]. Although SUMOylation has been thought to antagonize proteasomal degradation of proteins, the discovery of SUMO-targeted ubiquitin ligases (StUbls) has shown that SUMO and ubiquitin can collaborate to promote protein degradation [47]. StUbls are present at PML bodies [48] so it is likely that an as yet unidentified StUbl regulates BMAL1 SUMOylation and degradation.

## 3. Extranuclear SUMOylation

### 3.1. AMPA Receptors, Arc/Arg3.1 and SUMOylation–a Possible Pathway for Induction of LTP and Synaptic Scaling

The activity-dependent removal and insertion of AMPA receptors from and into the post-synaptic membrane underlies long-term depression (LTD) and long-term potentiation (LTP) [49,50]. Although AMPA receptors themselves do not appear to be SUMO substrates [19], synaptic SUMOylation has been implicated in LTP [51]. For example, glycine-induced chemical LTP leads to an increased co-localization of Ubc9 and SUMO1 in dendrites [52], suggesting activity-dependent loading of Ubc9 with SUMO. A possible SUMO target known to regulate AMPA receptor surface expression is the activity-related cytoskeletal-associated protein Arc/Arg3.1 [18]. It has been suggested that SUMOylation causes Arc to relocate into dendrites [18] where interaction of Arc with the cytoskeleton is involved in the establishment and maintenance of LTP and synaptic scaling [7,18].

### 3.2. SUMOylation in Agonist-Induced Endocytosis and Plasticity of Kainate Receptors

Kainate receptors (KARs) play important roles in the regulation of synaptic transmission and neuronal excitability [53,54,55,56]. The KAR subunit GluK2 is SUMOylated in response to agonist stimulation, which leads to endocytosis of GluK2-containing kainate receptors [19]. Although both, kainate and NMDA-stimulation, lead to KAR internalisation, NMDA-induced endocytosis does not involve GluK2 SUMO modification [19]. Agonist-induced or NMDA-induced endocytosis events are respectively associated with GluK2 degradation or recycling [57]. Thus, it is tempting to speculate that SUMOylation might target GluK2 towards lysosomal degradation following endocytosis, whereas non-modified GluK2 is recycled and can be subsequently re-inserted into the membrane. 

**Figure 2:**
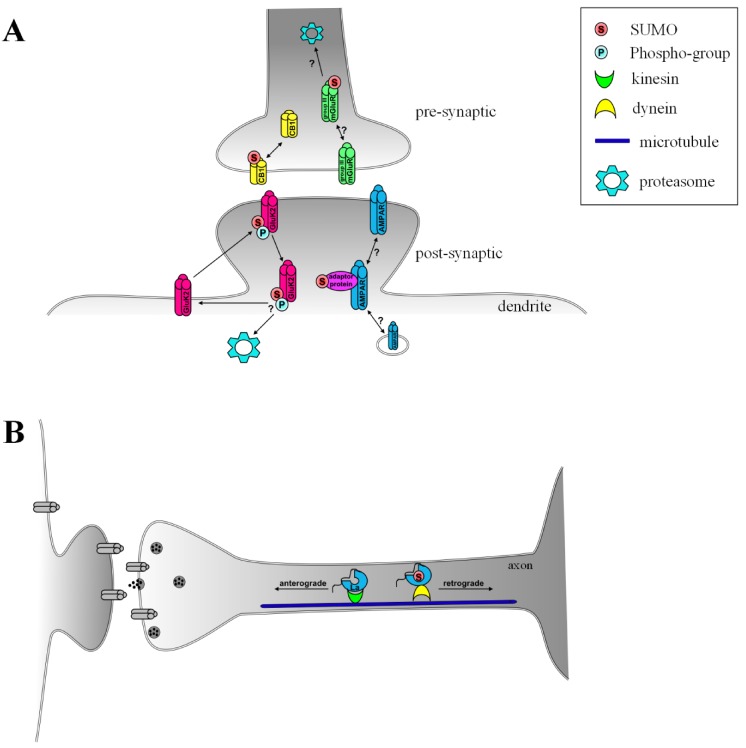
Influence of SUMOylation on receptor trafficking and protein transport. (**A**) At the pre-synapse, de-SUMOylation of the cannabinoid receptor CB1 is proposed to lead to its internalization. In contrast, SUMOylation of group III mGluRs may lead to internalisation and/or degradation. Agonist-induced phosphorylation of GluK2 in post-synaptic kainate receptors causes endocytosis. Potential SUMO regulation of AMPA receptors may be via SUMO modified interacting proteins; (**B**) In the axon, the RNA binding protein La is SUMOylated, triggering its retrograde axonal transport through binding to dynein. Abbreviations: mGluR: metabotropic glutamate receptor.

More recently, details of how SUMOylation of GluK2 is regulated and the importance of this modification for KAR plasticity have emerged. It has been shown that kainate stimulation leads to PKC-mediated phosphorylation of GluK2 which, in turn, promotes its SUMOylation and internalization [58]. Notably, this phosphorylation-mediated SUMOylation of GluK2 is required for the removal of GluK2-containing KARs during KAR LTD at mossy fibre synapses [59], providing the first direct example of a requirement for SUMO-mediated protein trafficking in a form of synaptic plasticity (Figure 2a).

### 3.3. Group III Metabotropic Glutamate Receptors–Genuine SUMO Targets?

The group III family of metabotropic glutamate receptors (mGluRs) comprises mGluR4 and mGluR6-8 [60]. They are expressed throughout the brain and, with the exception of mGluR6, are mostly presynaptic at glutamatergic and GABAergic terminals, where they act to regulate presynaptic release [60]. In yeast two-hybrid assays the c-termini of mGluR8a and 8b interact with Ubc9 and SUMO1, in addition to the SUMO E3 ligases PIAS1, PIASγ, PIASxβ. Subsequently, it was shown that PIAS1 interacts with the c-termini of all group III mGluRs [21] and that the mGluR8 c-terminus can also interact with the E3 ligases Pc2 and PIAS3L [20]. In addition, full-length mGluR8b can be SUMOylated in HEK293 cells co-transfected with SUMO1 [20].

However, despite these data, no direct co-immunoprecipitation of SUMOylated endogenous mGluRs from neurons or brain has yet been achieved and the functional consequences of mGluR SUMOylation remain to be determined. Because of this it has been questioned if group III mGluRs are actually true SUMO targets. For example, although mGluR7 is SUMOylated at a specific lysine residue *in vitro*, SUMO modification in neurons was not detected and no functional or trafficking differences were observed upon over-expression of a non-SUMOylatable mutant of mGluR7 [61]. Thus, while it remains possible that endogenous mGluRs do undergo SUMOylation, the number of receptors SUMOylated at any given time may be very small and the functional consequences very subtle.

### 3.4. G-Protein Coupled Cannabinoid Receptor 1

The cannabinoid receptor 1 (CB1) is the most widely-expressed G-protein coupled receptor in the brain [62]. CB1 is distributed largely presynaptically and, upon agonist activation, acts to suppress neuronal excitability and neurotransmitter release through coupling to G_i_ and G_o_ G proteins [63]. In primary rat cortical neurons the CB1 agonist Δ9-THC has been reported to increase levels of un-conjugated SUMO1, an effect blocked by the selective CB1 antagonist AM251. The authors propose that some of this increase in free SUMO1 derives from de-SUMOylation of CB1 and the tumor suppressor p53 following treatment with Δ9-THC [22] and that de-SUMOylation of CB1 causes its internalization while de-SUMOylation of p53 hinders its nuclear export [22,64]. Further validation is required but these observations would suggest that CB1 is unusual in being highly SUMOylated under basal conditions, in contrast to the vast majority of reported SUMO substrates, and that its trafficking and translocation are induced by activity-dependent deSUMOylation.

### 3.5. SUMOylation of La–Determining the Direction of Transport on the Microtubule Network

It is well-established that local protein translation occurs in axons and dendrites [65]. This requires the packaging of mRNAs and components of the translation machinery into granules and transport to designated translation sites [66]. The RNA-binding protein La recognizes RNA transcripts containing a 5’-UTR terminal oligopyrimidine (TOP) element and acts as an RNA chaperone during transport [67]. Neurons express several mRNAs containing the TOP motif, including the known La target grp78/BiP [68]. 

La can move along the microtubule network in retrograde or anterograde directions by binding dynein or kinesin, respectively [23]. SUMOylation of La appears to be the switch that determines the direction of La transport. SUMOylated La only binds to dynein and therefore is subject to retrograde transport towards the nucleus. Conversely, non-SUMOylated La binds kinesin and undergoes anterograde transport [23]. This is an intriguing observation and further work should define if SUMOylation might act as a general switch that can determine the polarity of target protein transport (Figure 2b).

## 4. SUMOylation in Cytoplasm–Nuclear Transport

### 4.1. SUMOylation of Glycogen Synthase Kinase 3 β (GSK3β)–Translocation to the Nucleus

The serine/threonine kinase GSK3β plays a central role in many cell pathways including energy metabolism, Wnt signalling, neuronal development, inflammation, tumorigenesis and cell death [69,70,71,72]. In neurons, GSK3β has been reported to be involved in regulating the balance between LTP and LTD [73] and has been implicated in a number of neurodegenerative disorders. Like many of the proteins it phosphorylates, GSK3β can be SUMOylated [13]. Wild type GSK3β is present throughout the cytoplasm and nucleus whereas a SUMOylation-deficient GSK3β mutant is excluded from the nucleus and present only in the cytoplasm [13]. It remains to be established if deSUMOylated GSK3β is actively exported from the nucleus or if the SUMO moiety acts as a nuclear localization signal. Whatever the mechanism, it appears again that SUMOylation acts as a switch to bring about translocation/re-localization of the substrate protein, potentially playing a central role in synaptic plasticity and neuronal function.

### 4.2. SUMO-Associated Nuclear Shuttling of Focal Adhesion Kinase (FAK)

FAK is an important mediator of signals between the extracellular matrix and the cytoplasm and is involved in controlling cell motility, shape and adhesion [74,75]. In neurons, FAK is important for neuronal migration and axon pathfinding [76,77,78]. FAK autophosporylates at Tyr397 to create a binding site for interaction partners including other kinases such as Src and Fyn [79]. SUMOylation of FAK at Lys152 increases autophosphorylation and promotes its nuclear localization [17,80]. However, the nuclear function of FAK remains unclear and further work is required to define how this nucleo-cytoplasmic shuttling of FAK is mediated.

### 4.3. Caspase SUMOylation Functions as a Nuclear Localization Signal

The caspase family of cysteine proteases mediate apoptotic neuronal death [81] but members of this family have also been reported to play physiological roles in AMPA receptor trafficking and synaptic plasticity [82]. In general, caspases can be divided into two subgroups, namely the initiator caspases and the effector caspases. Members of both subgroups have been reported to be SUMOylated, which promotes their nuclear localization [14,15,16]. Caspase-2 and caspase-7 are localized in nuclear speckles, identified as PML bodies for caspase-2 [15,16], whereas caspase-8 shows a more dispersed nuclear distribution [14]. It has been hypothesized that this translocation of caspases to the nucleus acts on specific targets in circumstances of cell stress such as neuronal hypoxia in the brain [15]. Interestingly, among the caspases there is no common domain containing the putative SUMOylation site. For the initiator caspases the SUMOylation sites are located within the death effector domain or the caspase recruitment domain [14,16] but the SUMOylation site of the effector caspase-7 is within the p20 subunit of the activated caspase [15]. As yet, very little functional data has been reported but SUMOylation of caspase-2 appears to increase its maturation from procaspase-2 [16]. Clearly, there is plenty of potential for future investigation.

## 5. Conclusions

Protein SUMOylation is a major regulator of neuronal function and dysfunction. It is involved in many diverse cell pathways and, in many cases transient SUMOylation initiates the translocation of substrate proteins between compartments within the cell. Misregulation of these SUMO-regulated processes can disrupt proper protein translocation and has been implicated in a wide range of neuronal diseases. Thus, for these substrates, SUMOylation can be viewed as a mobilization factor that orchestrates appropriate protein–protein interactions to mediate relocation and instigate downstream functional consequences. As we have pointed out, understanding of the mechanisms, targets and consequences of SUMOylation in neurons is at a very early stage. It is clear, however, that this is an important and exciting field that will undoubtedly shed new light on fundamental aspects of neuronal function and dysfunction.
